# Metyrapone single administration, as a possible predictive tool of its dosage and timing in Cushing’s syndrome

**DOI:** 10.3389/fendo.2024.1511155

**Published:** 2024-12-23

**Authors:** Yasutaka Tsujimoto, Naoki Yamamoto, Hironori Bando, Masaaki Yamamoto, Yuka Ohmachi, Yuma Motomura, Yuka Oi-Yo, Yuriko Sasaki, Masaki Suzuki, Shin Urai, Michiko Takahashi, Genzo Iguchi, Wataru Ogawa, Hidenori Fukuoka

**Affiliations:** ^1^ Division of Diabetes and Endocrinology, Department of Internal Medicine, Kobe University Graduate School of Medicine, Kobe, Japan; ^2^ Division of Diabetes and Endocrinology, Department of Internal Medicine, Kobe University Hospital, Kobe, Japan; ^3^ Department of Nutrition, Kobe University Hospital, Kobe, Japan; ^4^ Department of Clinical Nutrition and Dietetics, Konan Women’s University, Kobe, Japan

**Keywords:** Cushing’s syndrome, Cushing’s disease, metyrapone, hypercortisolemia, drug responsiveness, monitoring marker

## Abstract

Metyrapone is commonly used in the initial management of Cushing’s syndrome to reduce hypercortisolemia, but its optimal dosage and timing can vary significantly between patients. Currently, there are limited guidelines on adjustment methods for its administration to individual needs. This study aimed to evaluate responsiveness of each patient to metyrapone and identify the patient characteristics associated with the indices of cortisol responsiveness following a low-dose metyrapone. This single-center retrospective observational study included 15 treatment-naïve patients, 7 of whom had Cushing’s disease and 8 had adrenal Cushing’s syndrome. Serum cortisol levels were measured hourly from the time of administration of 250 mg of metyrapone up to four hours afterward. Parameters analyzed included the nadir of serum cortisol levels (F_nadir_), the difference between basal and nadir serum cortisol levels (_Δ_F), the time to nadir, and the characteristics of the patients. As a result, cortisol suppression curves showed significant variability among patients, particularly in the time to nadir. While the median time to nadir was 2 hours, 20% of patients required 4 hours or more, and these responses were not associated with patient characteristics. F_nadir_ was positively correlated with early-morning serum cortisol levels, serum cortisol levels after low-dose dexamethasone suppression test (LDDST), and urinary free cortisol (UFC) levels, whereas _Δ_F was positively correlated with late-night serum cortisol levels, serum cortisol levels after LDDST, and UFC levels. In conclusion, the duration of response to metyrapone appeared unpredictable in patients with Cushing’s syndrome and did not correlate with patient characteristics at baseline. Tracking the effect of metyrapone following a single low-dose administration may explain this variability and provide insights for optimizing individual dosing regimens. Further studies are required to validate these findings and guide more personalized treatment adjustments.

## Introduction

In patients with Cushing’s syndrome, hypercortisolemia causes various complications, including cerebrocardiovascular events, infectious diseases, and thromboembolisms, which increase mortality (1–3). The first-line treatment for this syndrome is to surgically remove the causative tumor if it is accessible (4, 5). However, pharmacotherapy is an option when surgery is not indicated or curative, or when hypercortisolemia is severe and treatment needs to be initiated before surgery (4, 5).

Adrenal steroidogenesis inhibitors such as metyrapone and osilodrostat are drugs that reliably suppress cortisol among medical treatments for this syndrome (4, 6–8). Metyrapone exhibits a short half-life, and its efficacy for the treatment of Cushing’s syndrome has long been established (7). A meta-analysis showed that the estimated percentage of cortisol normalization was 75.9% (9). In addition, the first prospective multicenter trial of metyrapone in patients with Cushing’s syndrome showed 47% remission at week 12 and improvements in physical, symptomatic, and metabolic profiles (10). Therefore, in countries where it is available, metyrapone is often used as the first-line treatment for Cushing’s syndrome, especially in the initial phase.

However, some clinical challenges remain in adjusting the metyrapone regimen for treating Cushing’s syndrome. First, no indicators exist for predicting responsiveness while introducing or titrating metyrapone. Normalization of 24-hour urinary free cortisol (UFC) (4), morning serum cortisol (6, 11), daily curve of serum cortisol (11), mean value of daily serum cortisol (12), and salivary cortisol levels (13) have been proposed as indicators for therapeutic monitoring. However, no independent indicators have been established. In particular, limited literature describes changes in cortisol levels following initiation or dose titration of metyrapone and corresponding adjustments (12). In one study, serum cortisol levels were monitored hourly before and after introducing metyrapone over four hours. Cortisol levels decreased within 2 h and the effect was sustained at 4 h. However, the factors associated with reactivity to metyrapone have not been identified (12). Second, life-threatening events can arise from the abrupt reduction of hypercortisolemia following metyrapone intake in Cushing’s syndrome. Excessive suppression of hypercortisolemia by metyrapone causes adrenal insufficiency (11, 12) and glucocorticoid withdrawal syndrome (14). Furthermore, the combination of metyrapone-induced adrenal insufficiency and physical stress, such as an infection, can lead to a health-related emergency (15, 16). In particular, *Pneumocystis jirovecii* pneumonia (PCP) may occur in patients with severe Cushing’s syndrome. It is a serious complication occurring in HIV patients, but it has also exhibited high mortality rates(35–50%) in immunocompromised host patients without HIV, including those with Cushing’s syndrome (17–19). Importantly, a significant proportion of patients with Cushing’s syndrome who developed PCP had greater reductions in cortisol prior to the event and not an adverse event of adrenal steroidogenesis inhibitors itself (17). Therefore, in severe cases with Cushing’s syndrome, gradual normalization of hypercortisolemia using trimethoprim-sulfamethoxazole prophylaxis may be recommended (4). These findings suggested that in the medical management of Cushing’s syndrome, the regimen should be tailored based on individual responsiveness to metyrapone.

Identifying factors that predict cortisol responsiveness would be beneficial in optimizing treatment. This study aimed to document the responsiveness to metyrapone and elucidate patient factors associated with changes in cortisol levels. A single 250 mg dose of metyrapone was administered, and cortisol levels were measured hourly for four hours. Furthermore, whether this administration could predict the responsiveness to metyrapone, specifically in terms of the degree of cortisol suppression and the duration of its inhibition, was also investigated.

## Materials and methods

### Study population

This was a retrospective, single-center, observational study. Patient information was used from the database, which was created because we considered the multipoint measurement of cortisol to be a valid method of response to metyrapone (13). Patients with treatment-naïve Cushing’s syndrome were admitted to Kobe University Hospital between May 2020 and April 2024. These patients were observed to have cortisol responses based on the protocol of metyrapone single administration described in the next section. Among some patients with Cushing’s disease and adrenal Cushing’s syndrome, this method could not be used for varying circumstances, such as excessively severe hypercortisolemia, absence of clinical symptoms related to hypercortisolemia, or scheduling conflicts for both the patient and the physician. Metyrapone was administered according to the protocol modifying the previous study as described in the next section (12). In all patients, the diagnosis, including the subtype of Cushing’s syndrome, was based on established guidelines (1, 20). These patients were treated with 0.5 mg or 1 mg dexamethasone overnight for the low-dose dexamethasone suppression test (LDDST) for diagnosis (20, 21). Furthermore, all patients were considered suitable for treatment with metyrapone based on the comprehensive agreement of our department, including those that required pre-operative medical treatment because of developing symptoms or those with high perioperative risk due to hypercortisolemia. Patients with ectopic adrenocorticotropic hormone syndrome were excluded because this test was not performed due to poor general condition.

In all patients, the following items were collected as patient background characteristics before metyrapone initiation: age, sex, height, weight, body mass index (BMI), body surface area (BSA); hepatic function parameters including platelet count, aspartate aminotransferase, and alanine aminotransferase; renal function parameters including serum creatinine and estimated glomerular filtration rate (eGFR); early-morning and late-night serum cortisol levels, serum cortisol levels after LDDST, UFC levels, and subtypes of Cushing’s syndrome. UFC levels were measured by 24-hour urine collection and they are expressed as multiples of the upper limit of normal (ULN). None of the patients had concurrent hepatic or renal failure. The study adhered to the Declaration of Helsinki and was approved by the Research Ethics Committee of Kobe University Hospital (B230127). Written informed consent was obtained from all patients.

### The protocol of metyrapone single administration

Metyrapone was administered to all patients early in the morning during hospitalization. Initially, the patients underwent intravenous line placement to collect blood samples. After resting in the supine position for 30 min, the patients were orally administered a test dose of metyrapone (Metopiron capsule^Ⓡ^; Ceolia Pharma, Tokyo, Japan). Subsequently, serial samples were collected before and at 1, 2, 3, and 4 h after administration. Although the sampling protocol followed a previously established method (12), the test dose was modified from 750 mg to 250 mg to enhance safety and reduce the risk of adverse effects. This adjustment was made to improve the feasibility of a single administration in routine clinical use, particularly in patients with varying degrees of hypercortisolemia.

**Figure 1:**
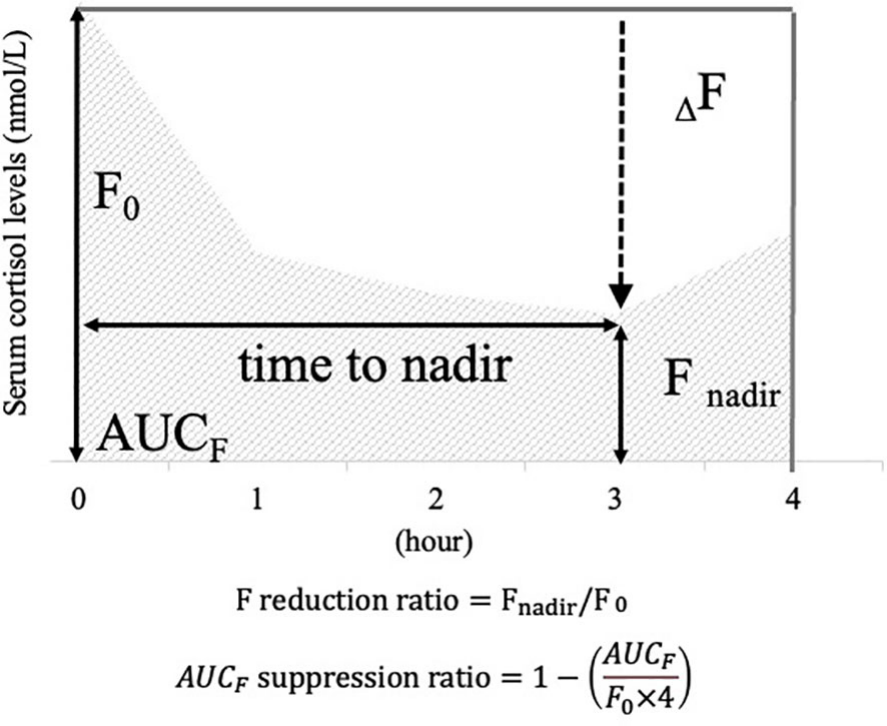
Items defined as cortisol responsiveness indices after the metyrapone single administration. To illustrate the definition of each item, a scheme for serum cortisol response after a single dose of metyrapone is provided. Vertical axis: serum cortisol levels; horizontal axis: hours after metyrapone administration. For illustrative purposes, this figure shows an example in which the nadir occurs at 3 h. In actual cases, the time to nadir varies among patients. F_0_: serum cortisol value at 0 hour; F_nadir_: minimum value of serum cortisol after administration; _Δ_F: difference between F_0_ and F_nadir_; F reduction ratio: ratio calculated by dividing _Δ_F by F_0_; AUC_F_: area under the curve plotted with serum cortisol values at each time point.

Serum cortisol levels were measured at each interval, and cortisol curves were plotted for each case, with serum cortisol on the vertical axis and time on the horizontal axis. The following items were defined as indicators to assess responsiveness to metyrapone (**Figure 1**): “F_nadir_” was defined as the nadir value of serum cortisol levels after metyrapone administration. “_Δ_F” was calculated as the difference between F_nadir_ and “F_0_”, which was defined as the serum cortisol at zero h, ( FΔ =F0−Fnadir ). “F reduction ratio” was calculated by dividing _Δ_F by F_0_. The area under the curve with F (“AUC_F_”) was calculated by applying the trapezoid rule to serum cortisol increments between 0 and 4 h, which was the cumulative sum of serum cortisol levels at each time point. “AUC_F_ suppression ratio” was calculated as one minus the AUC_F_ divided by (F_0_ × four), which is the ratio of decrease in AUC_F_ from the AUC if F_0_ continues for 4 h ( $AUC_{F}\;suppression\;ratio=1-\left(\frac{AUC_{F}}{F_{0}\times 4}\right)$ ). Additionally, the “time to nadir” was defined as the time at which F_nadir_ was measured. These indicators are defined based on previous studies (22, 23).

Subsequently, the metyrapone dosage and timing were adjusted based on the consensus of our department team members, to normalize UFC levels and reduce the complications caused by hypercortisolemia. Adverse events related to metyrapone were retrospectively investigated by reviewing medical records.

### Hormone assays

Serum cortisol levels were measured by an enzyme immunoassay (Electro Chemiluminescence Immunoassay and Enzyme Immunoassay; TOSOH, Tokyo, Japan, RRID: AB_3099658). The reference range of early morning cortisol was 162.7–468.9 nmol/L (5.9–17.0 μg/dL). The assay shows minimal cross-reactivity with other glucocorticoids. UFC levels were measured using an immunoradiometric assay (IRMA; TFB, Tokyo, Japan, RRID: AB_2894408) or chemiluminescent immunoassay (CLIA; Siemens, Tokyo, Japan, RRID: AB_2893154). The reference range of UFC measured by CLIA was 15.1–184.0 nmol/day (5.5–66.7 μg/day). Therefore, the UFC levels measured by IRMA were corrected to the values measured by CLIA using the following formula: (X=(Y+2.80)/0.917, X = CLIA, Y = IRMA).

### Statistical analysis

Data are presented as medians and interquartile ranges (IQR). The correlations between each metyrapone responsiveness index and patient background characteristics before metyrapone initiation described in Study population section were calculated. The correlation between nonparametric data was assessed using Spearman’s rank correlation test on a continuous scale and the Kruskal–Wallis test on a nominal scale. Numerical data between the two groups was compared using the Mann-Whitney *U* test. For categorical data, Fisher’s exact test was used to calculate the odds ratios. An analysis of covariance (ANCOVA) was conducted to adjust for covariates. *p* < 0.05 was considered statistically significant. All statistical analyses were performed using JMP 17.0 (JMP Statistical Discovery LLC, NC, USA).

## Results

### Patient characteristics

During the inclusion period, among the 30 patients with Cushing’s disease and adrenal Cushing’s syndrome were identified, this low-dose metyrapone administration was implemented in 15 patients. Among the 15 patients, 13 (87%) were women, 7 (47%) had Cushing’s disease, and 8 (53%) had adrenal Cushing’s syndrome. The median age, BMI, and BSA were 51.7 (39.0 – 64.0) years, 26.5 (22.6 – 30.8) kg/m^2^, and 1.6 (1.5 – 1.8) m^2^, respectively. The early-morning serum cortisol levels, serum cortisol levels after LDDST, and ULN of UFC were 604.1 (506.2 – 634.4) nmol/L, 593.0 (562.7 – 682.7) nmol/L, and 6.1 (3.7 – 4.5) nmol/day, respectively, which is consistent with active Cushing’s syndrome (**Table 1**). The patients did not have significant hepatic or renal failure. (**Supplementary Table 1**). Significant hepatic disease was defined as liver enzyme levels more than five times ULN or the presence of liver cirrhosis. Significant renal disease was defined as an eGFR less than 30 mL/min/1.73 m² or the need for dialysis. In these patients, single-dose metyrapone was administered to monitor responsiveness followed by the initiation of metyrapone as preoperative medical therapy.

**Table 1:** Patient characteristics.

Characteristics	All (n =15)	Cushing’s disease (n =7)	Adrenal Cushing’s syndrome (n =8)	*p* value
age, y.o.	51.7 (39.0 – 64.0)	61.0 (54.0 – 65.5)	39.0 (33.5 – 54.0)	0.049
sex	F/M 13/2	F/M 6/1	F/M 7/1	1.0†
height, m	1.5 (1.5 – 1.6)	1.5 (1.4 – 1.5)	1.5 (1.5 – 1.6)	0.41
body weight, kg	65.3 (52.4 – 74.8)	60.0 (48.9 – 74.5)	72.7 (59.0 – 74.3)	0.48
BMI, kg/m^2^	26.5 (22.6 – 30.8)	25.8 (22.2 – 29.9)	25.6 (23.2 – 30.9)	0.64
BSA, m^2^	1.6 (1.5 – 1.8)	1.5 (1.4 – 1.7)	1.7 (1.5 – 1.8)	0.56
early morning serum cortisol, nmol/L	604.1 (506.2 – 634.4)	642.7 (622.0 – 839.9)	535.1 (486.2 – 571.0)	0.024
late-night serum cortisol, nmol/L	556.2 (437.2 – 649.6)	664.8 (506.2 – 815.1)	528.2 (451.7 – 567.5)	0.13
serum cortisol after LDDST, nmol/L	593.0 (562.7 – 682.7)	706.2 (573.7 – 1246.8)	580.6 (515.8 – 621.3)	0.11
UFC, fold-change of ULN	6.1 (3.7 – 14.5)	16.5 (5.1 – 19.6)	4.89 (3.8 – 6.2)	0.083

Data are presented as median (IQR).

Each parameter for the groups of Cushing’s disease and adrenal Cushing’s syndrome was tested using the Mann-Whitney U test.

† For the categorical data, a Fisher's exact test was used to calculate the odds ratio and p value. The odds ratio was 0.85.

BMI, body mass index; BSA, body surface area; LDDST, low-dose dexamethasone suppression test; UFC, urinary free cortisol;

ULN, multiples of the upper limit of normal; F, female; M, male; IQR, interquartile range.

### Tracking cortisol responsiveness to the metyrapone single administration

**Figure 2:**
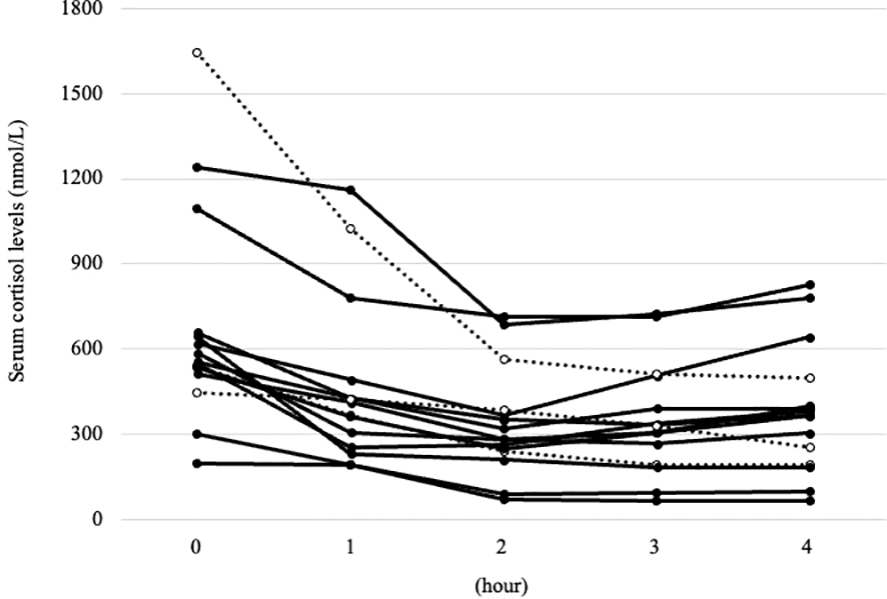
The cortisol curves for each case after the metyrapone single administration. Solid line: cortisol curves for patients with time to nadir of less than four hours, dotted line: cortisol curve for patients with time to nadir of four hours or more.

The cortisol curves varied among patients, especially in the time to nadir and _Δ_F (**Figure 2**). The time to nadir in each case was 1 (n =1), 2 (n =7), or 3 h (n =4), with a median of 2 h. Notably, in 20% (n =3) of patients, the time to nadir was estimated to be 4 h or more. The median values for each index of the cortisol responsiveness, F_nadir_, _Δ_F, F reduction ratio, and AUC_F_ suppression ratio were 264.8 (219.4 – 347.6) nmol/L, 295.2 (241.4 – 367.9) nmol/L, 0.52 (0.44 – 0.66) and 0.39 (0.28 – 0.44), respectively, indicating that metyrapone demonstrated a certain effect to suppress the cortisol level in all patients (**Table 2**). _Δ_F in Cushing’s disease was higher than that in adrenal Cushing’s syndrome, whereas the time to nadir and other cortisol responsiveness indicators did not differ between the subtypes (**Table 2**). Adverse events related to metyrapone were not reported in any patient.

**Table 2:** Each indicator value of cortisol responsiveness after the metyrapone single administration.

Indicators	All (n =15)	Cushing’s disease (n =7)	Adrenal Cushing’s syndrome (n =8)	*p* value
F_nadir_, nmol/L	264.8 (219.4 – 347.6)	364.1 (255.1 – 593.0)	253.7 (208.2 – 268.9)	0.08
_Δ_F, nmol/L	295.2 (241.4 – 367.9)	383.4 (302.0 – 506.2)	267.5 (205.5 – 297.2)	0.03
F reduction ratio	0.52 (0.44 – 0.66)	0.44 (0.41 – 0.67)	0.52 (0.49 – 0.57)	0.48
AUC_F_ suppression ratio	0.39 (0.28 – 0.44)	0.27 (0.27 – 0.49)	0.39 (0.35 – 0.41)	0.81
time to the nadir, hour	2.0 (2.0 – 3.0)	2.0 (2.0 – 3.5)	2.5 (2.0 – 3.0)	0.75

Data were presented as median (IQR).

IQR, interquartile range.

### Relationship between patient background characteristics and cortisol responsiveness indicators after the metyrapone administration

As anticipated, F_nadir_ was positively correlated with early morning serum cortisol levels (*r_s_* = 0.71, *p* < 0.01), serum cortisol levels after the LDDST (*r_s_* = 0.58, *p* = 0.02), and UFC levels (*r_s_* = 0.76, *p* < 0.01). However, _Δ_F was positively correlated with late-night serum cortisol levels (*r_s_* = 0.92, *p ≤* 0.01), serum cortisol levels after LDDST (*r_s_* = 0.82, *p* ≤ 0.01), and UFC levels (*r_s_* = 0.52, *p* = 0.047) (**Table 3**). Similarly, _Δ_F in the Cushing’s disease group was correlated with late-night serum cortisol levels (*r_s_* = 0.92, *p* ≤ 0.01) and serum cortisol levels after the LDDST (*r_s_* = 0.78, *p* = 0.03), whereas no such correlation was observed in the adrenal Cushing’s syndrome group (**Supplementary Table 2**). ANCOVA was conducted to determine the independent effects of these three levels on F_nadir_, with age as a covariate. The results positively correlated F_nadir_ with early morning cortisol (F = 4.92, *p* = 0.046) and UFC levels (F = 9.64, *p* = 0.009). However, no significant association was found between F_nadir_ and serum cortisol levels after the LDDST (F = 3.30, *p* = 0.094). Age did not influence this association in any of the models. Although high doses of metyrapone are generally used in cases of severe hypercortisolemia, this finding suggests that higher autonomous cortisol secretion is unexpectedly associated with increased cortisol reactivity to low-dose metyrapone. Factors correlated with the F reduction ratio, AUC_F_ suppression ratio, and time to nadir were not detected among the examined patient background characteristics. Factors such as sex, BMI, BSA, and hepatic and renal functions were not associated with any of the indicators (**Supplementary Table 3**).

**Table 3:** The correlations between patient background factors and the indicators after the metyrapone single administration.

	early morning serum cortisol levels		late-night serum cortisol levels		serum cortisol levels after LDDST		UFC		age		BMI		BSA	
	*r_s_*	*p* value	*r_s_*	*p* value	*r_s_*	*p* value	*r_s_*	*p* value	*r_s_*	*p* value	*r_s_*	*p* value	*r_s_*	*p* value
F_nadir_	0.71	<0.01	0.47	0.08	0.58	0.02	0.76	<0.01	0.62	0.01	0.22	0.42	0.06	0.83
_Δ_F	0.51	0.05	0.92	<0.01	0.82	<0.01	0.52	0.04	0.35	0.20	-0.18	0.53	-0.27	0.33
F reduction ratio	-0.27	0.33	0.21	0.44	0.05	0.85	-0.15	0.59	-0.3	0.28	-0.38	0.16	-0.18	0.51
AUC_F_ suppression ratio	-0.04	0.88	0.27	0.34	0.18	0.51	-0.02	0.95	-0.19	0.50	-0.16	0.56	-0.04	0.88
time to nadir	-0.01	0.97	0.31	0.27	0.23	0.42	-0.04	0.88	0.11	0.69	-0.48	0.07	-0.5	0.06

BMI, body mass index; BSA, body surface area; LDDST, low-dose dexamethasone suppression test; UFC, urinary free cortisol.

## Discussion

Our findings exhibit that cortisol responsiveness to metyrapone varies among patients with Cushing’s syndrome. For effective management of hypercortisolemia, it is important to predict drug responsiveness in each patient. The present data demonstrate that although the decline in cortisol levels may be greater in patients with higher basal cortisol levels, the time to nadir is unpredictable at baseline. Hence, tracking the response to metyrapone administration before initiation may enable better adjustments in drug dosage and timing to optimize the personalized medicine of Cushing’s syndrome.

The time to nadir was revealed by tracking the response of a single administration of metyrapone, which may predict the duration of the effect. The time to nadir was 2 h in the majority of patients, which was consistent with the brief half-life of metyrapone and a previous study, where a larger dosage of metyrapone was used (12). In such patients, to normalize the circadian rhythm of cortisol and UFC, it may be more effective to increase the frequency and adjust the timing of metyrapone administration rather than increasing the dose. Our previous study demonstrated that modifying the timing and frequency of metyrapone administration based on multiple measurements of salivary cortisol could lead to normalization (13). However, our present data demonstrated that in 20% of the patients, the time to nadir was four hours or more. In these patients, the effect may be prolonged, and increasing the dosage may lead to adrenal insufficiency. Therefore, it may not be necessary to increase the frequency of administration. No factors in patient background characteristics in this study were correlated with the time to nadir. Determining the time and frequency of daily metyrapone administration can be challenging without tracking responsiveness to a single administration.

Notably, the measures of responsiveness to metyrapone, such as _Δ_F and F_nadir_, did not correlate with parameters of body size or hepatic and renal function. Therefore, only tracking cortisol responsiveness to metyrapone can confirm cortisol reactivity and guide dosage adjustments to gradually normalize hypercortisolemia. Interestingly, each indicator of cortisol hypersecretion in Cushing’s syndrome demonstrated positive correlations with _Δ_F, suggesting that the extent of cortisol suppression by metyrapone is unexpectedly greater in cases with a more severe manifestation. Additionally, _Δ_F in patients with Cushing’s disease was greater than that in those with adrenal Cushing’s syndrome. However, this might be attributed to differences in early morning serum cortisol levels or variations in the mechanisms of the effect of metyrapone between these subtypes. The initiation of high-dose metyrapone is generally considered for patients with more severe hypercortisolemia (14, 17). However, if the reduction in cortisol levels by the drug is rapid and excessive, there is concern that it may lead to glucocorticoid withdrawal syndrome or life-threatening immune reconstitution inflammatory syndrome (IRIS), potentially resulting in PCP (4). In addition, our data revealed that a smaller F_nadir_ were correlated with lower values of each indicator of autonomous cortisol secretion, namely early morning serum cortisol levels, serum cortisol levels after LDDST and UFC. This suggests a possibility of adrenal insufficiency and confirms that F_nadir_ may be effective in preventing adrenal insufficiency in cases of Cushing’s syndrome with relatively mild cortisol excess.

This tracking cortisol responsiveness to the metyrapone single administration may be beneficial in determining the frequency and timing of dosing by assessing the time to nadir. In addition, because _Δ_F may be large at low doses even when the basal cortisol level is high, regardless of body size or hepatic and renal function, determining the single dosage by observing _Δ_F and F_nadir_ may be considered. For example, as shown in one of the cases in **Figure 2**, if the test dose significantly and briefly decreased serum cortisol levels from 535 (basal) to 248 nmol/L (nadir at 2 h), increasing the frequency without altering the dosage may be advisable. In fact, in this case, metyrapone at 250 mg three times a day successfully normalized UFC levels from 5 times the ULN to 0.9 times the ULN without adrenal insufficiency.

Our study had several limitations. Given the rarity of the disease and a single-center retrospective observational study, it was conducted with a limited number of patients. Additionally, as this study involved drug therapy in clinical practice, it was difficult to repeatedly administer metyrapone and perform blood sampling on individual cases to assess the reproducibility of its effects. However, similar to previous reports (12), the effects of metyrapone were reproducibly observed even with a low-dose in this study, and a more detailed evaluation was achieved for each case. To ensure an adequate number of patients, the analysis included patients with both Cushing’s disease and adrenal Cushing’s syndrome. However, it should be considered that the number of cases in each group was small. These two pathologies may have distinct cortisol secretion profiles, and their responsiveness to metyrapone may vary. Since this method was not applied to all patients during the study period, there may have been a selection bias. In this study, patients with severe hepatic disease, kidney disease, or gastrointestinal disorders were excluded; therefore, it is unclear whether this administration would be equally effective in these populations. Furthermore, to the best of our knowledge, there is no information on whether these conditions would affect the efficacy or duration of metyrapone action. To overcome these limitations, further prospective studies with larger sample sizes are required.

In conclusion, our study revealed that patients with higher autonomous cortisol secretion exhibited a stronger suppressive effect on cortisol following single-dose metyrapone administration. Additionally, the duration of metyrapone-induced cortisol suppression varied among patients and could not be predicted through patient characteristics. Consequently, metyrapone single dose administration before initiating therapy may be beneficial in determining the appropriate dosage and timing. Further studies are required to validate the usefulness of tracking cortisol responsiveness to metyrapone administration to optimize the metyrapone regimen.

## Data Availability

The raw data supporting the conclusions of this article will be made available by the authors, without undue reservation.
